# Supplementary material for: Association of Positive Bacterial Cultures Obtained from the Throat, Anus, Ear, Bronchi and Blood in Very-Low-Birth-Weight Premature Infants with Severe Retinopathy of Prematurity—Own Observations
Source: J Clin Med. 2023 Oct 5;12(19):6374. doi: 10.3390/jcm12196374 (PMC10573982; doi:10.3390/jcm12196374)
Supplement: Supplementary file 1 [file jcm-12-06374-s001.zip › jcm-2566392-supplementary.pdf]

Supplementary Table S1. Results of laboratory and morphology parameters of the examined groups of premature babies.

Time of measurement	Laboratory Parameters	CG<br>n=63	EG<br>n=51	p value
First 24h of life	**NLR (mean ± SD)**	1.5 ± 1.8	1.5 ± 1.6	0.62
	**WBC (mean ± SD)**<br>**> ULN**	11.3 ± 9.4<br>n = 7 (11%)	11.6 ± 6.6<br>n = 27 (53%)	0.31<br>**<0.001**
	**Neu**<br>**(mean ± SD), [%]**<br>**(mean ± SD), [G/l]**<br>**> ULN**	40 ± 18.6<br>5.3 ± 6.1<br>n = 23 (37%)	45.5 ± 19.2<br>5.5 ± 3.9<br>9 (18%)	0.13<br>0.04
	**Lymph**<br>**(mean ± SD), [%]**<br>**(mean ± SD), [G/l]**<br>**> ULN**	43.9 ± 18.5<br>4.8 ± 4.6<br>n = 22 (35%)	47.7 ± 22.9<br>5.3 ± 3.9<br>n = 13 (26%)	0.34<br>0.38
	**PLT (mean ± SD), [x10^{-3}/µl]**<br>**< LLN**	225.6 ± 70.6<br>n = 9 (14%)	190 ± 98.5<br>n = 20 (39%)	**<0.001**<br>**0.005**
	**CRP (mean ± SD), [mg/L]**<br>**>5 [mg/L]**	4.9 ± 11.6<br>n = 12 (19%)	6.7 ± 17.5<br>n = 12 (19%)	**0.01**<br>0.72
	**PRC (mean ± SD), [ng/ml]**<br>**>2 [ng/ml]**	5.8 ± 15.3<br>n = 23 (37%)	6 ± 9.5<br>22 (44%)	0.11<br>0.6
Second week of life[a]	**WBC (mean ± SD)**<br>**> ULN**	19.1 ± 11.8<br>n = 23 (37%)	20.2 ± 9.6<br>n = 9 (18%)	0.13<br>0.04
	**Neu**<br>**(mean ± SD), [%]**<br>**(mean ± SD), [G/l]**<br>**> ULN**	49.8 ± 15.7<br>47.3 ± 8.66<br>n = 38 (60%)	58.8 ± 15.4<br>12.4 ± 8.1<br>n = 12 (24%)	**<0.001**<br><0.001
	**Lymph**<br>**(mean ± SD), [%]**<br>**(mean ± SD), [G/l]**<br>**> ULN**	55 ± 15.5<br>10.8 ± 9.4<br>n = 28 (44%)	63.1 ± 15.5<br>12.9 ± 6.8<br>n = 48 (94%)	**<0.001**<br>**<0.001**
	**PLT (mean ± SD), [x10^{-3}/µl]**<br>**< LLN**	190.4 ± 70.4<br>n = 60 (95%)	132.5 ± 99.3<br>n = 40 (78%)	**<0.001**<br><0.02
	**CRP (mean ± SD), [mg/L]**<br>**>5 [mg/L]**	13.2 ± 22.2<br>n = 27 (43%)	28.4 ± 46.1<br>n = 38 (75%)	**<0.001**<br>**<0.001**
	**PRC (mean ± SD), [ng/ml]**<br>**>2 [ng/ml]**	14.4 ± 21.7<br>n = 42 (67%)	16 ± 23.4<br>n = 37 (73%)	0.66<br>0.64

[a]Mean (SD) for GA: EG 28 ± 1; Mean for CG 30 ± 1
Each parameter was calculated on the basis of mean values from given GA average period norms of CG and EG[1]:
ULN; upper limit of normal,
LLN; lower limit of normal,
P value is the statistically significant ratio of differences between laboratory parameters in EG vs. CG; Mann–Whitney test, Student's t-test, chi^2 Yates's correction for continuity
Statistically significant results for our work are marked in bold.
